# Deep learning-based assessment of PD-L1 expression in NSCLC predicts outcome for patients treated with anti-PD-1 immunotherapy

**DOI:** 10.3389/fimmu.2026.1750816

**Published:** 2026-02-13

**Authors:** Morgane Peroz, Nicolas Roussot, Alis Ilie, David Rageot, Valentin Derangere, Caroline Truntzer, François Ghiringhelli

**Affiliations:** 1 Université Bourgogne Europe, Centre Georges-François Leclerc, Unicancer, Cancer Biology Transfer Platform, UMR INSERM 1231, Therapies and Immune Response in Cancers (TIRECs) team, Dijon, France; 2 Department of Medical Oncology, Centre Georges-François Leclerc, Dijon, France; 3 Genetic and Immunology Medical Institute, Dijon, France

**Keywords:** biomarker, deep learning - artificial intelligence, histopathalogical, lung, predictive model

## Abstract

**Background:**

PD-L1 expression is widely used as a predictive biomarker for anti-PD-1 therapies in non-small cell lung cancer (NSCLC). However, its prognostic value remains controversial. Here, we investigated whether deep learning (DL) applied to PD-L1 immunohistochemistry (IHC) slides could identify histological patterns predictive of outcome in patients treated with anti-PD-1 therapy.

**Methods:**

We analyzed two independent NSCLC cohorts: MSK (n=182, training) and CGFL (n=108, validation). Tumor regions were manually annotated, tiled, stain-normalized, and processed through the UNI foundation model to extract deep features. Clustering of tiles from 10 extreme-outcome MSK cases identified histology-based subgroups. These were then applied to the remaining patients by projection and majority voting. Associations with progression-free survival (PFS) and overall survival (OS) were assessed. DL groups were integrated with clinical covariates in a multivariate model.

**Results:**

Clustering revealed two distinct DL-defined groups (DL^High^ vs. DL^Low^). In the MSK cohort, DL^High^ patients had significantly longer PFS than DL^Low^ (median 5.7 vs. 2.5 months; HR = 0.63, 95% CI 0.44–0.89; p=0.01). This prognostic value was independently confirmed in the CGFL cohort (median PFS 15.2 vs. 6.2 months; HR = 0.59, 95% CI 0.36–0.96; p=0.03). OS was numerically higher in DL^High^ patients but did not reach significance. DL classification correlated with higher PD-L1 tumor proportion score (TPS). Discordance between DL and TPS was observed, and the DL model further stratified outcomes among patients with TPS ≥50%. A combined model integrating DL groups with clinical variables improved prediction of PFS compared to clinical features alone (HR = 0.50, 95% CI 0.33–0.75; p<0.001 in MSK; HR = 0.54, 95% CI 0.31–0.91; p=0.02 in CGFL).

**Conclusions:**

Deep learning applied to PD-L1 IHC slides identifies reproducible histomorphological patterns associated with outcomes in anti-PD-1–treated NSCLC patients. This approach provides prognostic information beyond conventional PD-L1 scoring and enhances predictive accuracy when combined with clinical factors.

## Introduction

Immune checkpoint inhibitors (ICIs) targeting the programmed death 1 (PD-1) and programmed death-ligand 1 (PD-L1) axis have revolutionized the treatment landscape of advanced and metastatic non-small cell lung cancer (NSCLC), offering durable clinical benefit and improved survival outcomes for a subset of patients (1–4). Currently, these treatments are commonly used as first-line therapy as monotherapy or in combination with chemotherapy in patients without targetable oncogenic driver alterations (5). Despite these advances, only approximately 20–30% of patients receiving ICI monotherapy experience meaningful responses, highlighting the critical need for more accurate predictive biomarkers to guide patient selection and optimize therapeutic efficacy (6, 7).

Currently, the expression of PD-L1 protein, typically measured by immunohistochemistry (IHC) on tumor biopsies, serves as the unique standard biomarker to stratify patients for anti-PD-1/PD-L1 therapies (8). In particular, the decision to select treatment comprising immunotherapy alone or chemoimmunotherapy is mainly based on the assessment of PD-L1 status using Tumor Proportion Score (TPS). When TPS is above 50%, immunotherapy alone may be used instead of chemoimmunotherapy (9). However, PD-L1 expression is an imperfect indicator of response due to several limitations. First, technical variability arising from different antibody clones (e.g., 22C3, SP263, QR1), staining protocols, and interobserver interpretation can lead to inconsistent scoring (10). In addition, intratumoral PD-L1 expression is not only limited to tumor cells, but heterogeneity of PD-L1 expression and dynamic changes induced by prior treatments or the tumor microenvironment further complicate accurate assessment (11). Clinically, some patients with low or negative PD-L1 expression may respond to ICIs, whereas a significant proportion of patients with high PD-L1 levels do not achieve clinical benefit.

In this context, the emergence of artificial intelligence (AI) and deep learning approaches in computational pathology offers promising solutions to overcome these challenges. Deep convolutional neural networks can analyze whole-slide images and extract subtle histopathologic and spatial features beyond the capabilities of traditional microscopy (12, 13). These models have been demonstrated to provide reproducible and objective PD-L1 quantification across different assays and institutions (14–16). Moreover, deep learning algorithms can integrate information on tumor-infiltrating immune cells, tumor architecture, and stromal components that are critical determinants of immunotherapy response, but difficult to quantify manually (17, 18).

Building on these technological advances, our study aims to develop and externally validate a deep learning–based model for the assessment of PD-L1 expression in NSCLC. We hypothesize that this approach will not only refine the accuracy and consistency of PD-L1 scoring but will also improve the prediction of clinical outcomes for patients treated with anti-PD-1 immunotherapy compared to conventional methods.

## Methods

### Patient selection

The first cohort was a public dataset downloaded from SYNAPSE (https://www.synapse.org/Synapse:syn26722053) and recently published (19) comprising 182 patients. The inclusion criteria for this cohort were: patients with stage IV NSCLC who initiated treatment with anti-PD-(L)1 blockade therapy between 2014 and 2019 at the study institution, and who had a baseline CT scan, baseline PD-L1 IHC assessment and next-generation sequencing by MSK IMPACT. The second cohort comprised 108 NSCLC tumor biopsies collected between 2015 and 2024 in the Department of Pathology of the Georges François Leclerc Cancer Center in Dijon, France. The inclusion criteria for this cohort were: patients with stage IV NSCLC who initiated treatment with anti-PD-(L)1 blockade therapy between January 2017 and December 2023 at the study institution.

### Ethics committee approval

Only patients from whom informed consent was obtained were included in this retrospective study. The present study was approved by the CNIL (French national commission for data privacy) and the Georges François Leclerc Cancer Center (Dijon, France) local ethics committee, and was performed in accordance with the Helsinki Declaration and European legislation. This study falls within the scope of the biobanking authorization registered under the registration number AC-2014-2260.

### Histological staining

MSK Cohort: IHC was performed on 4-μm FFPE tumor tissue sections using a standard PD-L1 antibody (E1L3N; dilution 1:100, Cell Signaling Technologies) validated in the clinical laboratory at the study institution. Staining was performed using an automated immunostaining platform (Bond III, Leica) using heat-based antigen retrieval employing a high pH buffer (epitope retrieval solution-2, Leica) for 30 min. A polymeric secondary kit (Refine, Leica) was used for detection of the primary antibody.

CGFL Cohort: PD-L1 protein expression in tumor cells was assessed using immunohistochemistry with a ready-to-use PDL1 commercial kit with QR1 or 22C3 antibodies. Tonsil tissue served as positive control tissue.

### Image digitalization

MSK Cohort: PD-L1 IHC-stained diagnostic slides were digitally scanned at a minimum of ×20 magnification using an Aperio Leica Biosystems GT450 v.1.0.0.

CGFL Cohort: PD-L1 IHC-stained diagnostic slides were digitalized with an Evident VS200 (Evident) at 20× magnification to generate a whole slide imaging (WSI) file in vsi format.

### Image analysis procedure

**Figure 1:**
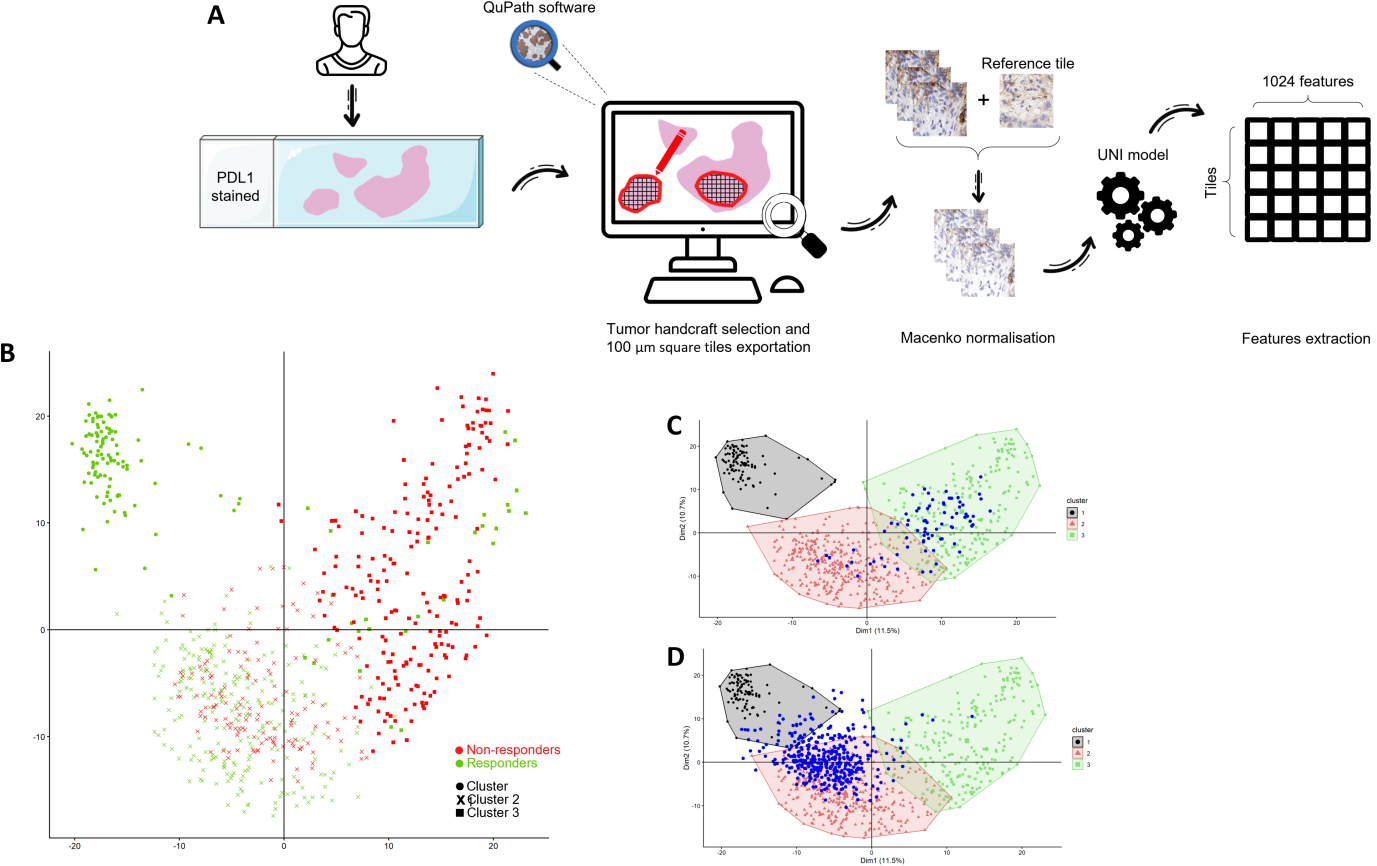
Feature analysis and cluster creation. **(A)** Flowchart of study design from PDL1 staining to UNI feature extraction. **(B)** Principal component analysis (PCA) of tile-level features extracted from 5 responder and 5 non-responder MSK patients. PCA was computed using all tiles from these patients (n=681), followed by hierarchical clustering on principal components (HCPC), resulting in three clusters. Different shapes indicate cluster membership, while tile color reflects response status (green: responders; red: non-responders). The numbers of tiles per cluster were 100, 349, and 232, respectively. Tiles from one MSK patient **(C)** and one CGFL patient **(D)** were projected a posteriori onto the PCA space trained on the MSK cohort without re-estimating either the PCA loadings or the cluster structure. Colored regions correspond to the convex hull enclosing all MSK tiles belonging to each cluster and are used solely to visualize the spatial extent of each group. Blue dots indicate tiles from the projected patient.

For all tumor slides, tumor area zones were manually selected, then these areas were separated into 100µm square tiles. Colors were normalized using MACENKO algorithm (20) and processed using UNI deep learning model (21) to extract high dimensional feature vectors (**Figure 1A**).

To ensure consistency across datasets, we performed feature-wise normalization using the MSK cohort as a reference. For each of the 1,024 features, we calculated its mean and standard deviation across all MSK patients. These feature-specific statistics were then used to normalize the data in the CGFL cohort: for each patient and each feature, the corresponding MSK cohort mean was subtracted and the result divided by the corresponding MSK cohort standard deviation. This procedure ensures that each feature is scaled relative to its distribution in the MSK cohort.

### Statistical analysis

Quantitative variables are described as median and Interquartile Range (IQR), and qualitative variables as number and percentage. Patient characteristics were compared by cohort (whole cohort, MSK and CGFL) using the Chi-2 or Fisher’s exact test for qualitative variables, and the Wilcoxon rank sum test for continuous variables, as appropriate.

Survival analysis was performed using the survival R library. The prognostic value of the different variables was tested using univariate or multivariate Cox models for PFS when conditions of the model validity were applicable. Proportional hazards assumptions were tested based on Schoenfeld residuals. When the proportionality assumption was not verified, we fitted an extended Cox model, with time dependent coefficients for relevant variables; the time varying coefficient was described with a parametric time function. Survival probabilities were estimated using the Kaplan–Meier method and survival curves were compared using the log-rank test when appropriate. When the proportional hazards assumption was not checked, the estimated restricted mean survival time (RMST) for DFS at 24 months was assessed to compare groups of interest (SurvRM2 R library (22)). P-values less than 0.05 were considered statistically significant.

Statistical analyses were performed using the R software (http://www.R-project.org/) and graphs were drawn using GraphPad Prism version 9.0.2.

## Results

### Patient selection and characteristics

We used a public data set from patients treated for NSCLC at Memorial Sloan Kettering (MSK) Cancer Center and who received PD-(L)1-blockade-based therapy. These patients were treated between 2014 and 2019 (cohort characteristics are shown in **Table 1**). The second data set is constituted of patients treated in France for NSCLC at Center Georges Francois Leclerc between 2015 and 2024 with PD-(L)1-blockade-based therapy or chemoimmunotherapy; this cohort was used as a validation cohort. In the total population, there were more male than female patients. Most patients were smokers or former smokers, and the main histological type was adenocarcinoma. When pooling both cohorts, in first line, 212 patients were treated with anti PD-1-blockade-based therapy and 76 with chemoimmunotherapy. Immunotherapy was used in first line for 146 (66%) patients. PD-L1 TPS status is 0% for 66 patients, between 1 and 49% for 74 patients and greater than 50% for 150 patients.

**Table 1:** Patient clinical characteristics in MSK, CGFL and whole cohort.

Characteristics	*Whole cohort N=290*	*MSK N=182*	*CGFL N=108*	*P-value*	*Adjusted P-value*
Smoking status					
Never	26 (9.3%)	21 (12%)	5 (5.2%)		
Current	149 (53%)	114 (63%)	35 (36%)	<0.001	<0.001
Former	104 (37%)	47 (26%)	57 (59%)		
Unknown	11	0	11		
WHO performance status					
0	48 (17%)	26 (14%)	22 (22%)	<0.001	<0.001
1	196 (69%)	142 (78%)	54 (53%)		
2	35 (12%)	12 (6.6%)	23 (23%)		
3	4 (1.4%)	2 (1.1%)	2 (2.0%)		
4	1 (0.4%)	0 (0%)	1 (1.0%)		
Unknown	6	0	6		
Histological type					
Adenocarcinoma	209 (72%)	129 (71%)	80 (74%)	0.4	0.4
Squamous	53 (18%)	32 (18%)	21 (19%)		
Other	28 (9.7%)	21 (12%)	7 (6.5%)		
RECIST					
Complete Response	22 (10%)	5 (4.3%)	17 (17%)	<0.001	<0.001
Partial Response	73 (34%)	32 (28%)	41 (41%)		
Stable Disease	34 (16%)	20 (17%)	14 (14%)		
Progressive Disease	87 (40%)	59 (51%)	28 (28%)		
Unknown	74	66	8		
Treatment					
Immunotherapy (Anti PD1 alone)	142 (64%)	112 (96%)	30 (28%)	<0.001	<0.001
Chemoimmmunotherapy	76 (34%)	0 (0%)	76 (72%)		
Anti PD1 and anti CTLA4 combination	4 (1.8%)	4 (3.4%)	0 (0%)		
Unknown	68	66	2		
Line of therapy					
1	146 (66%)	40 (34%)	106 (100%)	<0.001	<0.001
>1	76 (34%)	76 (66%)	0 (0%)		
Unknown	68	66	2		
PDL1 (cutoff at 50%)					
<50	140 (48%)	86 (47%)	54 (50%)	0.7	0.7
≥50	150 (52%)	96 (53%)	54 (50%)		
PDL1 (cutoff at 1%)					
0	66 (23%)	51 (28%)	15 (14%)	0.006	0.007
≥1	224 (77%)	131 (72%)	93 (86%)		

N (%) : Median (IQR), Fisher’s exact test ; Pearson’s Chi-squared test; * *p*-values were adjusted using Benjamini–Hochberg FDR correction.

Comparison of the clinical variable between the two cohorts showed differences for all available characteristics, except for histological type and PD-L1 TPS status with a cutoff at 50%, thus demonstrating the substantial heterogeneity between the two data sets.

### Generation of the deep learning procedure

10 patients from the MSK cohort were then isolated to train the model. We selected the five patients with the longest Progression-Free Survival (PFS) who did not progress, and the five patients with the shortest PFS who progressed. This corresponds to 361 tiles associated with response and 350 tiles associated with absence of response. Using Principal Components Analysis (PCA) followed by Hierarchical Clustering, tiles were separated into 3 clusters. Cluster 1 was constituted of responders only, cluster 2 was a mixture of responders and non-responders and cluster 3 was enriched in non-responders (**Figures 1A, B**).

**Figure 2:**
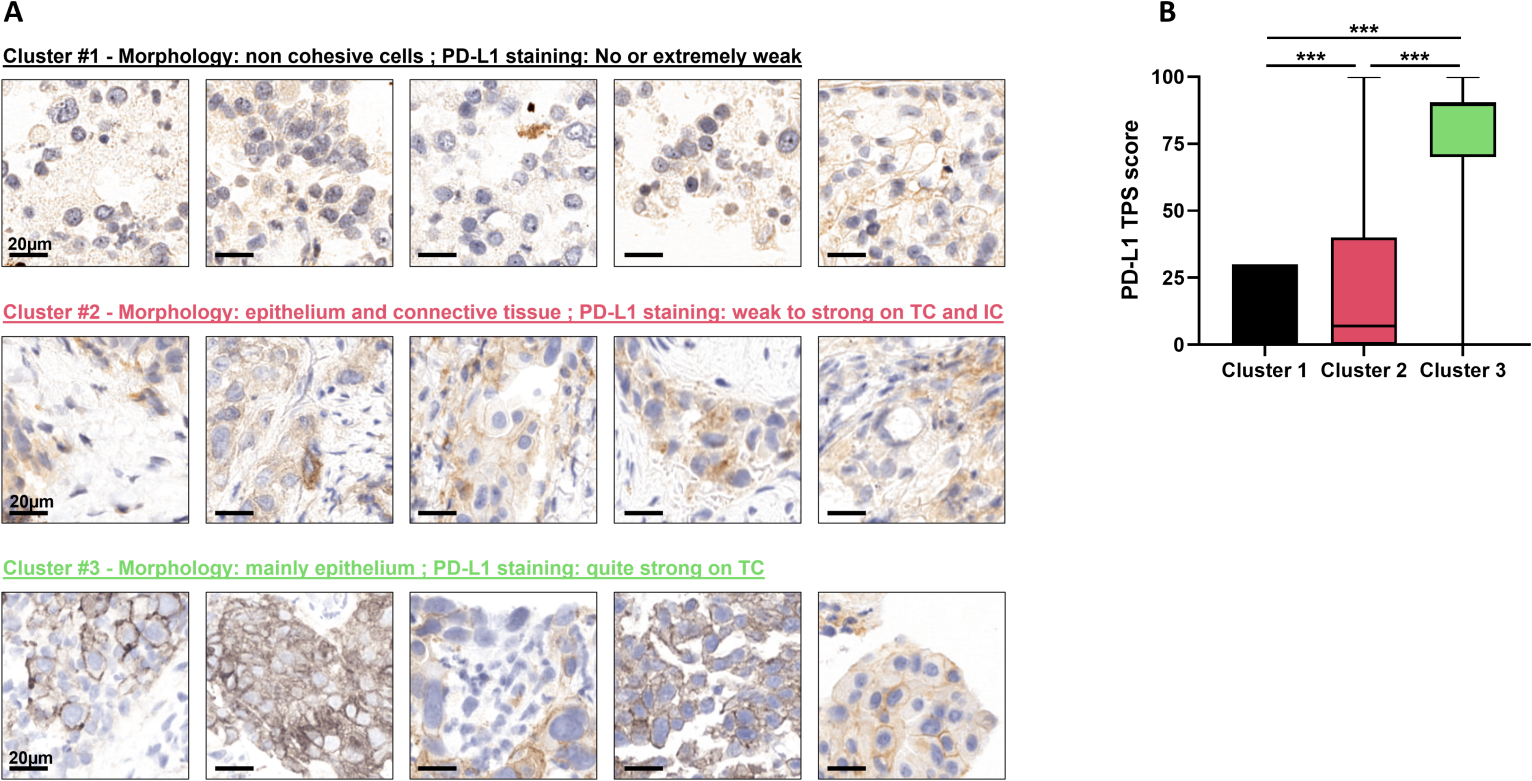
Cluster interpretation. **(A)** Representative tiles of each cluster with corresponding visual descriptions. **(B)** Boxplots of the PD-L1 TPS score in Clusters 1 (n=1923 tiles), 2 (n=21–193 tiles) and 3 (n=28–747 tiles) for the pooled cohort. ***p-value<0.001.

To illustrate which histological patterns distinguish DL^High^ from DL^Low^ groups, **Figure 2A** provides representative tiles of each cluster. Morphologically, Cluster 1 matched tiles with low-cohesive epithelial cells that displayed a negative or an extremely weak stain for PD-L1. Cluster 2 matched tiles that mixed tumor epithelial cells with or without adjacent connective tissue. In this cluster PD-L1 staining was either low or quite strong, localized on tumor cells (TC) or immune cells (IC). Finally, cluster 3 was mainly represented by tiles displaying epithelial tumor cells with strong PD-L1 staining.

These observations were concordant with quantitative evaluation of PD-L1 through staining (**Figure 2B**).

For the remainder of the patients, we projected each new patient’s tile onto the training PCA space (**Figures 1C, D**). We looked at which centroid this tile was closest to, and assigned it the label of the corresponding cluster. We then counted the total number of tiles assigned to each of the three clusters and, by majority voting, assigned the patient to the cluster with the most tiles. The same process was then applied in the remaining 172 patients from MSK cohort and on the validation set from 108 patients from CGFL (**Supplementary Figure S1**).

### Prognostic role of the deep learning model

**Figure 3:**
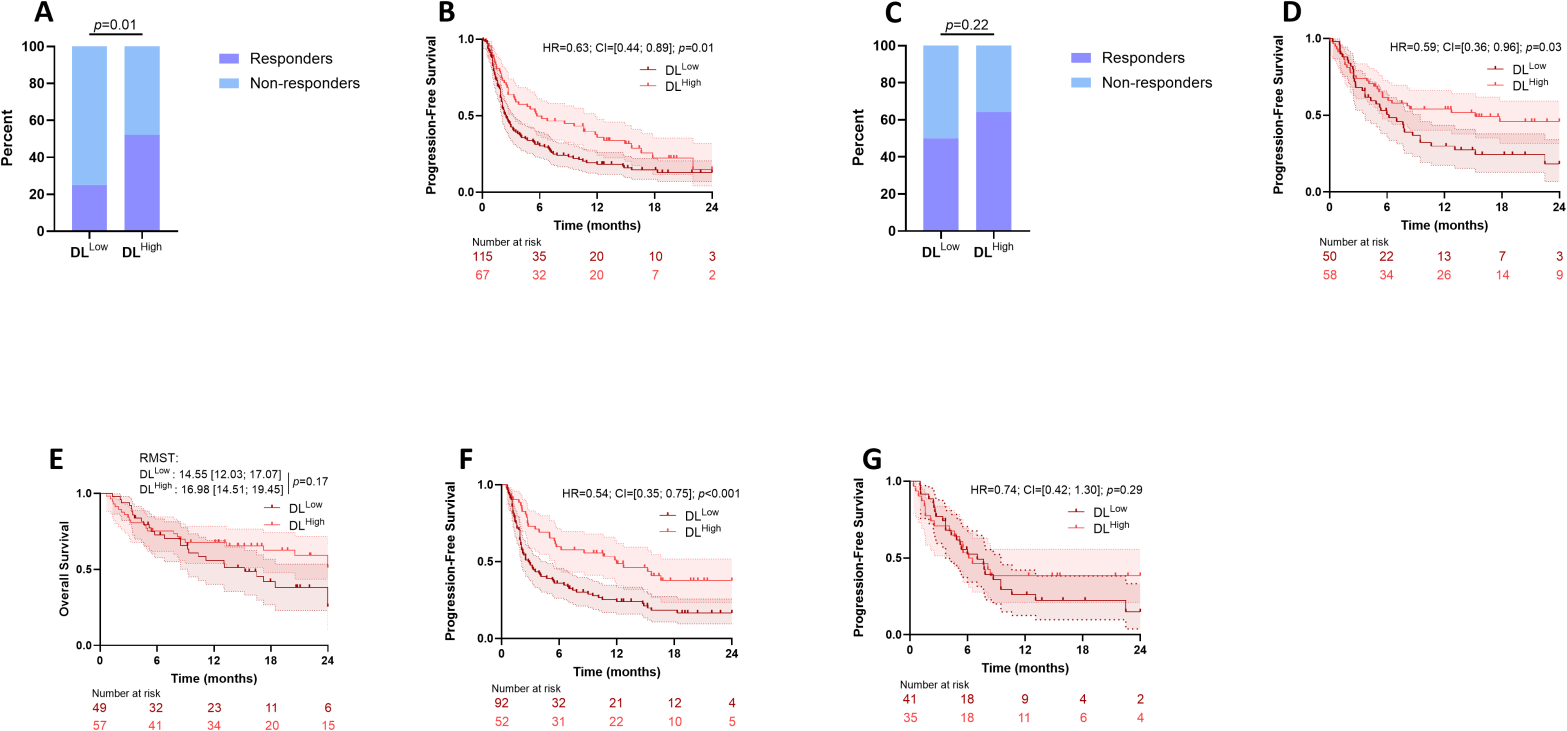
Association between survival and DL model derived groups. Barplots comparing the proportion of responders (Complete Response and Partial Response) and non-responders (Stable Disease and Progressive Disease) according to DL model derived classifier for the MSK **(A)** and the CGFL **(C)** cohorts. Kaplan-Meier curves with patients stratified according to the DL model derived classifier for progression-free survival for the MSK **(B)** and the CGFL **(D)** cohorts. **(E)** Kaplan–Meier curves with patients stratified according to the DL model derived classifier for overall survival for the CGFL cohort. Kaplan-Meier curves with patients stratified according to the DL model derived classifier for progression-free survival for the pooled cohort in patients treated with immunotherapy alone **(F)** and chemoimmunotherapy **(G)**. DL, Deep Learning.

Clusters 1 and 2 exhibited similar PFS rates (results not shown) and were thus grouped together: in the so-called DL^High^ group; cluster 3 constituted the DL^Low^ group. In the training set, 67 patients were attributed to the DL^High^ group and 115 patients to DL^Low^. When looking at response rates, there were 2 Complete Responses (CR), 14 Partial Responses (PR) in the DL^High^ group and 3 CR and 18 PR in the DL^Low^ group (Chi-2 test p-value=0.01). When using PFS as an endpoint, patients in the DL^High^ group had better PFS than patients classified as DL^Low^ (HR = 0.63 [0.44, 0.89; p=0.01) with a median PFS of 5.7 vs 2.5 months for training cohort. Overall survival was not available for this cohort(**Figures 3A, B**).

When applying the DL model in the validation cohort, 58 patients were attributed to the DL^High^ group and 50 patients to DL^Low^. When looking at response rates, there were 11 CR and 25 PR in the DL^High^ group, and 6 CR and 16 PR in the DL^Low^ group (Chi-2 test p-value =0.22). When using PFS as an endpoint, patients classified as DL^High^ had better PFS than patients classified as DL^Low^ (HR = 0.59 [0.36, 0.96]; p=0.03) with median PFS of 15.2 vs 6.2 months for the validation cohort. When looking at Overall Survival (OS), patients classified as DL^High^ did not have significantly better OS than patients classified as DL^Low^ (RMST: DL^Low^ 14.55[12.03;17.07] vs DL^High^ 16.98 [14.51;19.45]; *p* = 0.17) with median OS of 37.7 vs 15.2 months (**Figures 3C-E**).

To complete the analysis, all patients were grouped together and divided according to their treatment. The DL model successfully identified significant subgroups with distinct survival, offering a more refined stratification for patients treated with immunotherapy alone (**Figure 3F**). For patients treated with chemoimmunotherapy, the DL model did not distinguish patients’ outcome (**Figure 3G**).

### Correlation with PD-L1 TPS score

**Figure 4:**
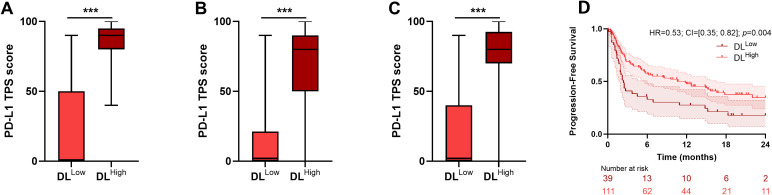
Link between PD-L1 TPS score and DL model derived groups. Boxplots of the PD-L1 TPS score in DL^Low^ and DL^High^ groups for MSK **(A)**, CGFL **(B)** and whole **(C)** cohorts. **(D)** Kaplan-Meier curves with patients stratified according to the DL model derived classifier for progression-free survival for the pooled cohort in the high PD-L1 TPS score group. ***p-value<0.001. DL, Deep Learning.

We examined the association between DL model groups and PD-L1 TPS score. In each cohort and in the pooled cohort, PD-L1 TPS score was significantly higher in patients in the DL^High^ group (**Figures 4A–C**). However, there was not complete agreement between the two scoring systems: PD-L1 TPS score 0% was detected in the DL^High^ group, while PD-L1 TPS score >50% were also detected in the DL^Low^ group.

Moreover, when stratifying patients into high (≥50%) and low (<50%) PD-L1 TPS score groups, the DL model successfully identified significant subgroups with distinct survival, offering a more refined stratification for patients with high PD-L1 TPS score (**Figure 4D**). In the low (<50%) PD-L1 TPS score group, the DL model did not significantly distinguish patients’ outcome (results not shown).

### DL score improves patient prediction in multivariate model

**Figure 5:**
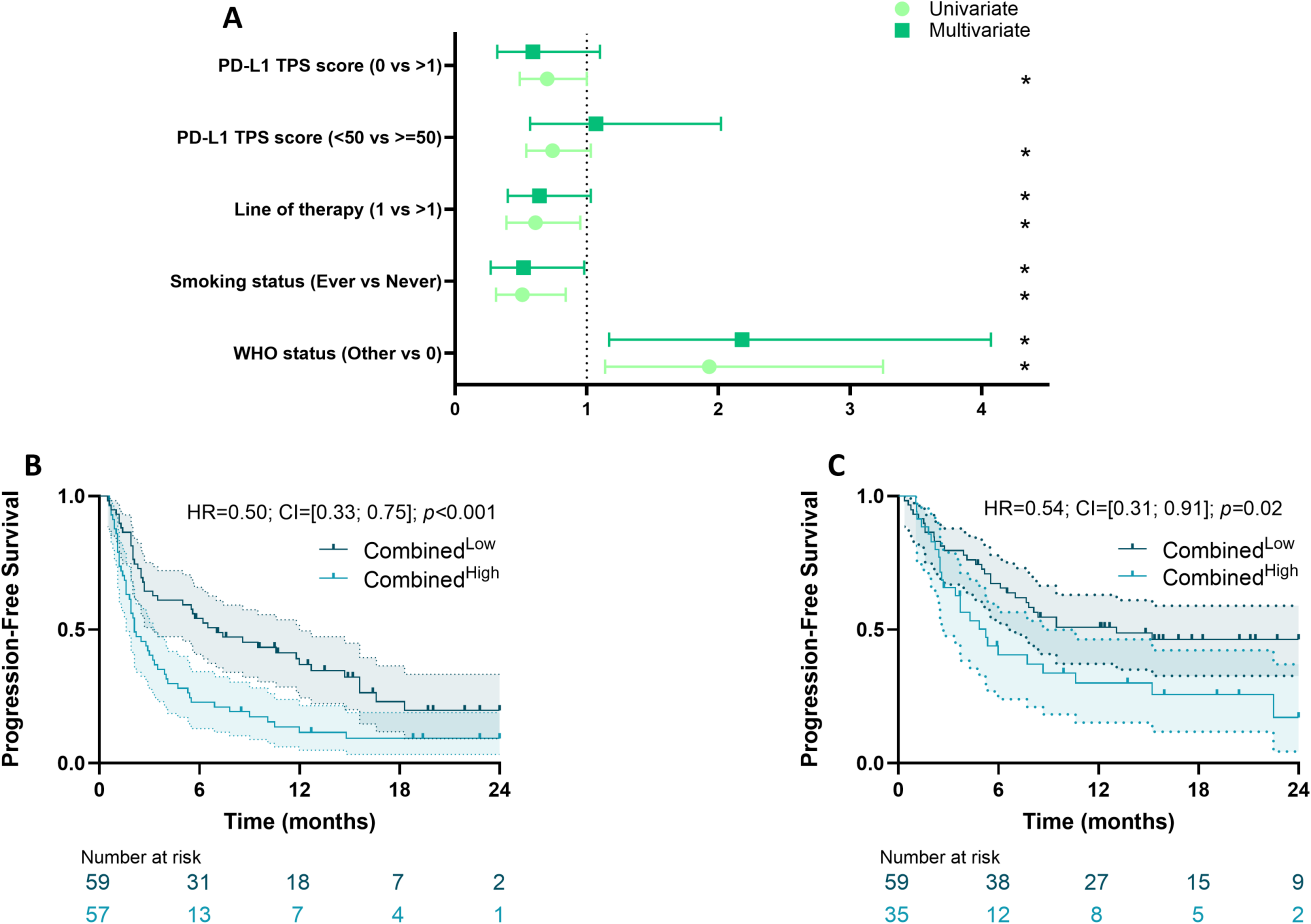
Survival analysis of clinical variables and DL model. **(A)** Forest plots representing hazard ratios and confidence intervals for univariate and multivariate Cox models for Progression-Free Survival estimated using clinical variables. *p-value<0.1. Kaplan-Meier curves with patients stratified according to the combined score for progression-free survival for the MSK **(B)** and the CGFL **(C)** cohorts. DL, Deep Learning.

Clinical variables associated with PFS were selected based on univariate Cox models, and a multivariate clinical model was then estimated based on variables with p-values<0.1 (**Figure 5A**; **Table 2**). Because PD-L1 TPS score is correlated with the DL model, this variable was excluded from the multivariate model. WHO performance status, smoking status, treatment information and line of therapy were retained in the model. Variables selected in the clinical model and the DL group variable were combined in a unique multivariate survival model, named the “combined model”. A combined score was then estimated using the linear predictor of the combined model. Using the median as a cut-off, patients with a low score had better PFS than those with a high score (HR = 0.50 [0.33; 0.75]; p<0.001, **Figure 5B**). Similar observations were made in the validation cohort, using a threshold adapted to the cohort (HR = 0.54 [0.31; 0.91]; p=0.02 (**Figure 5C**). In the pooled cohort, AUCs of the DL model, clinical and combined model were respectively 0.36, 0.66 and 0.71. The likelihood-ratio test showed that our DL score significantly added prognostic value to the clinical model (p=0.03 when comparing clinical and combined model).

**Table 2:** Univariate and multivariate Cox models for progression-free survival (PFS) in the MSK cohort. Only characteristics associated to PFS were reported.

		*Univariate*		*Multivariate*	
Characteristics	*N*	*HR (95% CI)*	*P-value*	*HR (95% CI)*	*P-value*
Smoking status	182				
Never					
Ever		0.51 (0.31; 0.84)	0.007	0.50 (0.27; 0.96)	0.036
WHO performance status	182				
0					
≥1		1.93 (1.14; 3.25)	0.014	2.10 (1.13; 3.90)	0.019
Histological type	182				
Adenocarcinoma					
Squamous		1.18 (0.78; 1.80)	0.4	1.29 (0.74; 2.26)	0.4
Other		0.80 (0.46; 1.40)	0.4	1.95 (0.90; 4.20)	0.089
PDL1 (cutoff at 50%)	182				
<50					
≥50		0.74 (0.54; 1.03)	0.076	0.79 (0.50; 1.26)	0.03
Treatment	116				
Immunotherapy					
Anti PD1 and anti CTLA4		2.99 (1.08; 8.27)	0.035	2.88 (1.03; 8.07)	0.045
Line of therapy	116				
>1					
1		0.61 (0.39; 0.95)	0.03	0.60 (0.37; 0.95)	0.03

HR, Hazard Ratio; CI, Confidence Interval.

## Discussion

The integration of ICIs into the treatment of advanced and metastatic NSCLC has transformed patient care by offering durable responses and improved survival for some patients (1–4). However, despite the revolutionary impact of agents targeting the PD-1/PD-L1 axis, the clinical benefit remains small and limited to approximately 20–30% of patients when allcomers are treated, a reflection of the underlying heterogeneity of NSCLC and the complex nature of antitumor immunity (7). This limitation underscores the critical need for reliable and robust biomarkers to optimize patient selection, guide therapeutic strategies, and ultimately enhance the efficacy of ICIs.

Current clinical decision-making relies heavily on the assessment of PD-L1 expression by IHC, with TPS guiding the choice between ICI monotherapy and chemoimmunotherapy (8, 9). While patients with high PD-L1 TPS (≥50%) may be offered immunotherapy alone, this biomarker is imperfect (10). As shown in recent reviews and practice guidelines, PD-L1 expression is subject to challenges such as technical variability among antibody clones and platforms, subjective interpretation, and spatial as well as temporal heterogeneity within tumors. Furthermore, discordance between PD-L1 status and response is well-documented: some patients with high PD-L1 expression achieve little clinical benefit, while others with low or undetectable PD-L1 respond to ICIs. These shortcomings have driven active research into alternative and complementary biomarkers, including circulating tumor DNA, tumor mutational burden, gene expression signatures, and features derived from the tumor microenvironment. However, the clinical utility of these emerging biomarkers remains under investigation, and none have yet supplemented PD-L1 in routine practice.

In this context, AI and deep learning technologies are emerging as powerful tools in computational pathology. By analyzing digitized histopathology slides, deep learning models can extract high-dimensional features beyond the limits of human interpretation, offering more objective, reproducible, and potentially more informative assessments of the tumor immune landscape. Some studies have established different deep learning models for evaluating or predicting PD-L1 and have shown strong explanatory and predictive power using either H&E or PD-L1 labeled IHC slides (23–30).

In addition, some reports support the capacity of deep learning models to predict outcome in NSCLC using H&E slides (31–34). The present study demonstrates the development and validation of a deep learning-based approach to assess PD-L1 expression and predict outcomes with anti-PD-1 therapy in NSCLC. Not only does the deep learning model provide more consistent scoring versus traditional IHC-based TPS, it also encapsulates critical contextual information such as spatial patterns of immune infiltration that are difficult to quantify manually, thus leading to improved prediction of prognosis in the group of patients with PD-L1 TPS score ≥50%. We assume that our deep learning approach makes it possible to add morphological information that is not taken into account by expression of PD-L1 protein alone.

The clinical utility of this approach is highlighted by its independent prognostic value in both the training and external validation cohorts. Notably, patients classified as DL^High^ by the model experienced significantly better progression-free and overall survival compared to the DL^Low^ group, outperforming conventional PD-L1 TPS for predicting RECIST response, as well as PFS and OS. Importantly, while a significant correlation between DL^High^ status and higher PD-L1 TPS was observed, there remained notable discordance, supporting the notion that deep learning captures complementary—and perhaps more clinically relevant—biological information. The value of the deep learning model in prognostic stratification was further confirmed for patients with high PD-L1 TPS.

These findings align with a growing body of literature advocating for the integration of digital pathology and machine learning into predictive biomarker development for immunotherapy response. AI models enabling clinically relevant risk stratification for cancer immunotherapy beyond conventional PD-L1 TPS have been proposed (31, 34). Some tools for mechanistic interpretability have been designed to extract interpretable spatial features from imaging data (34, 35). The ability of AI-driven models to standardize and enhance the interpretation of complex histological and immunological features represents a major step forward, potentially paving the way for more precise, individualized immunotherapy in lung cancer and beyond.

Nevertheless, several limitations of our study should be acknowledged. First, the choice to select 10 patients may be debated. This choice was intended to consider extreme patients as highlighting representative patterns of response. However, this does raise concerns about the generalizability of our model. Second, the manual annotation of tumor regions by pathologists is inherently subjective and may introduce observer-dependent bias. Third, the retrospective nature of the study, together with the relatively limited sample size used for model training, raises concerns about generalizability. Consequently, extensive validation in larger, prospective, and multi-institutional cohorts is warranted before definitive clinical translation can be considered.

Additionally, while the DL model was built on digitalized IHC slides for PD-L1, integration with other multi-omic and microenvironmental features—such as genomics, transcriptomics, and spatial immune profiling—may further improve predictive power and should be explored in future studies. Finally, future work could be performed to strengthen mechanistic interpretability of our DL model through quantification of tissue heterogeneity and organizational complexity (35).

In summary, this study provides compelling evidence that deep learning models applied to routine histopathology can overcome the technical and biological limitations inherent to traditional PD-L1 assessment, offering a pragmatic and scalable approach to refining immunotherapy selection in NSCLC. As the field moves toward increasingly data-driven and personalized cancer care, such innovations are poised to play a critical role in optimizing outcomes for patients receiving ICIs.

## Data Availability

The MSK cohort data is available at the following link https://www.synapse.org/Synapse:syn26722053 The CGFL cohort data are available under request.

## Supplementary material

Supplementary Figure 1 Workflow of the tile-based clustering and classification. From the MSK cohort (n=182), 10 patients (five responders and five non-responders) were used to perform Hierarchical Clustering on Principal Components (HCPC), identifying two IHC-based clusters. Tiles from the remaining MSK patients (n=172) and the independent CGFL cohort (n=108) were then projected onto this reference space and assigned to the closest cluster, allowing patient-level group prediction.

## References

[1] Brahmer J Reckamp KL Baas P Crinò L Eberhardt WEE Poddubskaya E . Nivolumab versus docetaxel in advanced squamous-cell non–small-cell lung cancer. New Engl J Med. (2015) 373:123–35. doi: 10.1056/NEJMoa1504627, PMID: 26028407 PMC4681400

[2] Borghaei H Paz-Ares L Horn L Spigel DR Steins M Ready NE . Nivolumab versus docetaxel in advanced nonsquamous non–small-cell lung cancer. New Engl J Med. (2015) 373:1627–39. doi: 10.1056/NEJMoa1507643, PMID: 26412456 PMC5705936

[3] Herbst RS Baas P Kim DW Felip E Pérez-Gracia JL Han JY . Pembrolizumab versus docetaxel for previously treated, PD-L1-positive, advanced non-small-cell lung cancer (KEYNOTE-010): a randomised controlled trial. Lancet. (2016) 387:1540–50. doi: 10.1016/S0140-6736(15)01281-7, PMID: 26712084

[4] Mok TSK Wu YL Kudaba I Kowalski DM Cho BC Turna HZ . Pembrolizumab versus chemotherapy for previously untreated, PD-L1-expressing, locally advanced or metastatic non-small-cell lung cancer (KEYNOTE-042): a randomised, open-label, controlled, phase 3 trial. Lancet. (2019) 393:1819–30. doi: 10.1016/S0140-6736(18)32409-7, PMID: 30955977

[5] Hendriks LE Kerr KM Menis J Mok TS Nestle U Passaro A . Non-oncogene-addicted metastatic non-small-cell lung cancer: ESMO Clinical Practice Guideline for diagnosis, treatment and follow-up. Ann Oncol. (2023) 34:358–76. doi: 10.1016/j.annonc.2022.12.013, PMID: 36669645

[6] Gandhi L Rodríguez-Abreu D Gadgeel S Esteban E Felip E Angelis FD . Pembrolizumab plus chemotherapy in metastatic non–small-cell lung cancer. New Engl J Med. (2018) 378:2078–92. doi: 10.1056/NEJMoa1801005, PMID: 29658856

[7] Mountzios G Remon J Hendriks LEL García-Campelo R Rolfo C Van Schil P . Immune-checkpoint inhibition for resectable non-small-cell lung cancer - opportunities and challenges. Nat Rev Clin Oncol. (2023) 20:664–77. doi: 10.1038/s41571-023-00794-7, PMID: 37488229

[8] Hirsch FR McElhinny A Stanforth D Ranger-Moore J Jansson M Kulangara K . PD-L1 immunohistochemistry assays for lung cancer: results from phase 1 of the blueprint PD-L1 IHC assay comparison project. J Thorac Oncol. (2017) 12:208–22. doi: 10.1016/j.jtho.2016.11.2228, PMID: 27913228

[9] Reck M Rodríguez-Abreu D Robinson AG . Pembrolizumab versus chemotherapy for PD-L1–positive non–small-cell lung cancer. N Engl J Med. (2016) 375:1823–33. doi: 10.1056/NEJMoa1606774, PMID: 27718847

[10] Büttner R Gosney JR Skov BG Adam J Motoi N Bloom KJ . Programmed death-ligand 1 immunohistochemistry testing: A review of analytical assays and clinical implementation in non-small-cell lung cancer. J Clin Oncol. (2017) 35:3867–76. doi: 10.1200/JCO.2017.74.7642, PMID: 29053400

[11] McLaughlin J Han G Schalper KA Carvajal-Hausdorf D Pelekanou V Rehman J . Quantitative assessment of the heterogeneity of PD-L1 expression in non–small-cell lung cancer. JAMA Oncol. (2016) 2:46–54. doi: 10.1001/jamaoncol.2015.3638, PMID: 26562159 PMC4941982

[12] Baxi V Edwards R Montalto M Saha S . Digital pathology and artificial intelligence in translational medicine and clinical practice. Mod Pathol. (2022) 35:23–32. doi: 10.1038/s41379-021-00919-2, PMID: 34611303 PMC8491759

[13] Coudray N Ocampo PS Sakellaropoulos T Narula N Snuderl M Fenyö D . Classification and mutation prediction from non-small cell lung cancer histopathology images using deep learning. Nat Med. (2018) 24:1559–67. doi: 10.1038/s41591-018-0177-5, PMID: 30224757 PMC9847512

[14] Hondelink LM Hüyük M Postmus PE Smit VTHBM Blom S von der Thüsen JH . Development and validation of a supervised deep learning algorithm for automated whole-slide programmed death-ligand 1 tumour proportion score assessment in non-small cell lung cancer. Histopathology. (2022) 80:635–47. doi: 10.1111/his.14571, PMID: 34786761 PMC9299490

[15] Huang Z Chen L Lv L Fu CC Jin Y Zheng Q . A new AI-assisted scoring system for PD-L1 expression in NSCLC. Comput Methods Programs Biomed. (2022) 221:106829. doi: 10.1016/j.cmpb.2022.106829, PMID: 35660765

[16] Shmatko A Ghaffari Laleh N Gerstung M Kather JN . Artificial intelligence in histopathology: enhancing cancer research and clinical oncology. Nat Cancer. (2022) 3:1026–38. doi: 10.1038/s43018-022-00436-4, PMID: 36138135

[17] Saltz J Gupta R Hou L Kurc T Singh P Nguyen V . Spatial organization and molecular correlation of tumor-infiltrating lymphocytes using deep learning on pathology images. Cell Rep. (2018) 23:181–193.e7. doi: 10.1016/j.celrep.2018.03.086, PMID: 29617659 PMC5943714

[18] Zhang J Choi H Kim Y Park J Cho S Kim E . Artificial intelligence-based digital pathology using H&E-stained whole slide images in immuno-oncology: from immune biomarker detection to immunotherapy response prediction. J Immunother Cancer. (2025) 13:e011346. doi: 10.1136/jitc-2024-011346, PMID: 40759439 PMC12323524

[19] Vanguri RS Luo J Aukerman AT Egger JV Fong CJ Horvat N . Multimodal integration of radiology, pathology and genomics for prediction of response to PD-(L)1 blockade in patients with non-small cell lung cancer. Nat Cancer. (2022) 3:1151–64. doi: 10.1038/s43018-022-00416-8, PMID: 36038778 PMC9586871

[20] Macenko M Niethammer M Marron JS Borland D Woosley JT Guan X . (2009). A method for normalizing histology slides for quantitative analysis, in: 2009 IEEE International Symposium on Biomedical Imaging: From Nano to Macro, . pp. 1107–10. doi: 10.1109/ISBI.2009.5193250

[21] Chen RJ Ding T Lu MY Williamson DFK Jaume G Song AH . Towards a general-purpose foundation model for computational pathology. Nat Med. (2024) 30:850–62. doi: 10.1038/s41591-024-02857-3, PMID: 38504018 PMC11403354

[22] Uno H Claggett B Tian L Inoue E Gallo P Miyata T . Moving beyond the hazard ratio in quantifying the between-group difference in survival analysis. J Clin Oncol. (2014) 32:2380–5. doi: 10.1200/JCO.2014.55.2208, PMID: 24982461 PMC4105489

[23] Ge C Shi Y Wang W Zhang A Huang M Zhao F . Artificial Intelligence-driven image analysis for standardised programmed death-ligand 1 expression evaluation in non-small cell lung cancer. Diagn Pathol. (2025) 20:1–12. doi: 10.1186/s13000-025-01707-1, PMID: 41013460 PMC12465877

[24] Shamai G Livne A Polónia A Sabo E Cretu A Bar-Sela G . Deep learning-based image analysis predicts PD-L1 status from H&E-stained histopathology images in breast cancer. Nat Commun. (2022) 13:6753. doi: 10.1038/s41467-022-34275-9, PMID: 36347854 PMC9643479

[25] Sha L Osinski BL Ho IY Tan TL Willis C Weiss H . Multi-field-of-view deep learning model predicts nonsmall cell lung cancer programmed death-ligand 1 status from whole-slide hematoxylin and eosin images. J Pathol Inform. (2019) 10:24. doi: 10.4103/jpi.jpi_24_19, PMID: 31523482 PMC6669997

[26] Herbst RS Prizant H Ruderman D Conway J Shamshoian J Koeppen H . Digital versus manual PD-L1 scoring in advanced NSCLC from the IMpower110 and IMpower150 trials. J Thorac Oncol. (2025) 20:1778–90. doi: 10.1016/j.jtho.2025.07.131, PMID: 40759194

[27] Wu L Wei D Chen W Wu C Lu Z Li S . Comprehensive potential of artificial intelligence for predicting PD-L1 expression and EGFR mutations in lung cancer: A systematic review and meta-analysis. J Comput Assist Tomogr. (2025) 49:101. doi: 10.1097/RCT.0000000000001644, PMID: 39143665

[28] Plass M Olteanu GE Dacic S Kern I Zacharias M Popper H . Comparative performance of PD-L1 scoring by pathologists and AI algorithms. Histopathology. (2025) 87:90–100. doi: 10.1111/his.15432, PMID: 39961605 PMC12129605

[29] Kim H Kim S Choi S Park C Park S Pereira S . Clinical validation of artificial intelligence–powered PD-L1 tumor proportion score interpretation for immune checkpoint inhibitor response prediction in non–small cell lung cancer. JCO Precis Oncol. (2024) 8):e2300556. doi: 10.1200/PO.23.00556, PMID: 38723233

[30] Molero A Hernandez S Alonso M Peressini M Curto D Lopez-Rios F . Assessment of PD-L1 expression and tumour infiltrating lymphocytes in early-stage non-small cell lung carcinoma with artificial intelligence algorithms. J Clin Pathol. (2025) 78:456–64. doi: 10.1136/jcp-2024-209766, PMID: 39419594 PMC12322406

[31] Rakaee M Tafavvoghi M Ricciuti B Alessi JV Cortellini A Citarella F . Deep learning model for predicting immunotherapy response in advanced non–small cell lung cancer. JAMA Oncol. (2025) 11:109–18. doi: 10.1001/jamaoncol.2024.5356, PMID: 39724105 PMC11843371

[32] Tourniaire P Ilie M Mazières J Vigier A Ghiringhelli F Piton N . WhARIO: whole-slide-image-based survival analysis for patients treated with immunotherapy. JMI. (2024) 11:037502. doi: 10.1117/1.JMI.11.3.037502, PMID: 38737491 PMC11088447

[33] Captier N Lerousseau M Orlhac F Hovhannisyan-Baghdasarian N Luporsi M Woff E . Integration of clinical, pathological, radiological, and transcriptomic data improves prediction for first-line immunotherapy outcome in metastatic non-small cell lung cancer. Nat Commun. (2025) 16:614. doi: 10.1038/s41467-025-55847-5, PMID: 39800784 PMC11725576

[34] Li X . Deciphering cell to cell spatial relationship for pathology images using SpatialQPFs. Sci Rep. (2024) 14:29585. doi: 10.1038/s41598-024-81383-1, PMID: 39609630 PMC11605059

[35] Li X Ren X Venugopal R . Entropy measures for quantifying complexity in digital pathology and spatial omics. iScience. (2025) 28:112765. doi: 10.1016/j.isci.2025.112765, PMID: 40546955 PMC12178799

