# Supplementary figures and images for: Deep learning-based assessment of PD-L1 expression in NSCLC predicts outcome for patients treated with anti-PD-1 immunotherapy
Source: Front Immunol. 2026 Feb 13;17:1750816. doi: 10.3389/fimmu.2026.1750816 (PMC12946059; doi:10.3389/fimmu.2026.1750816)

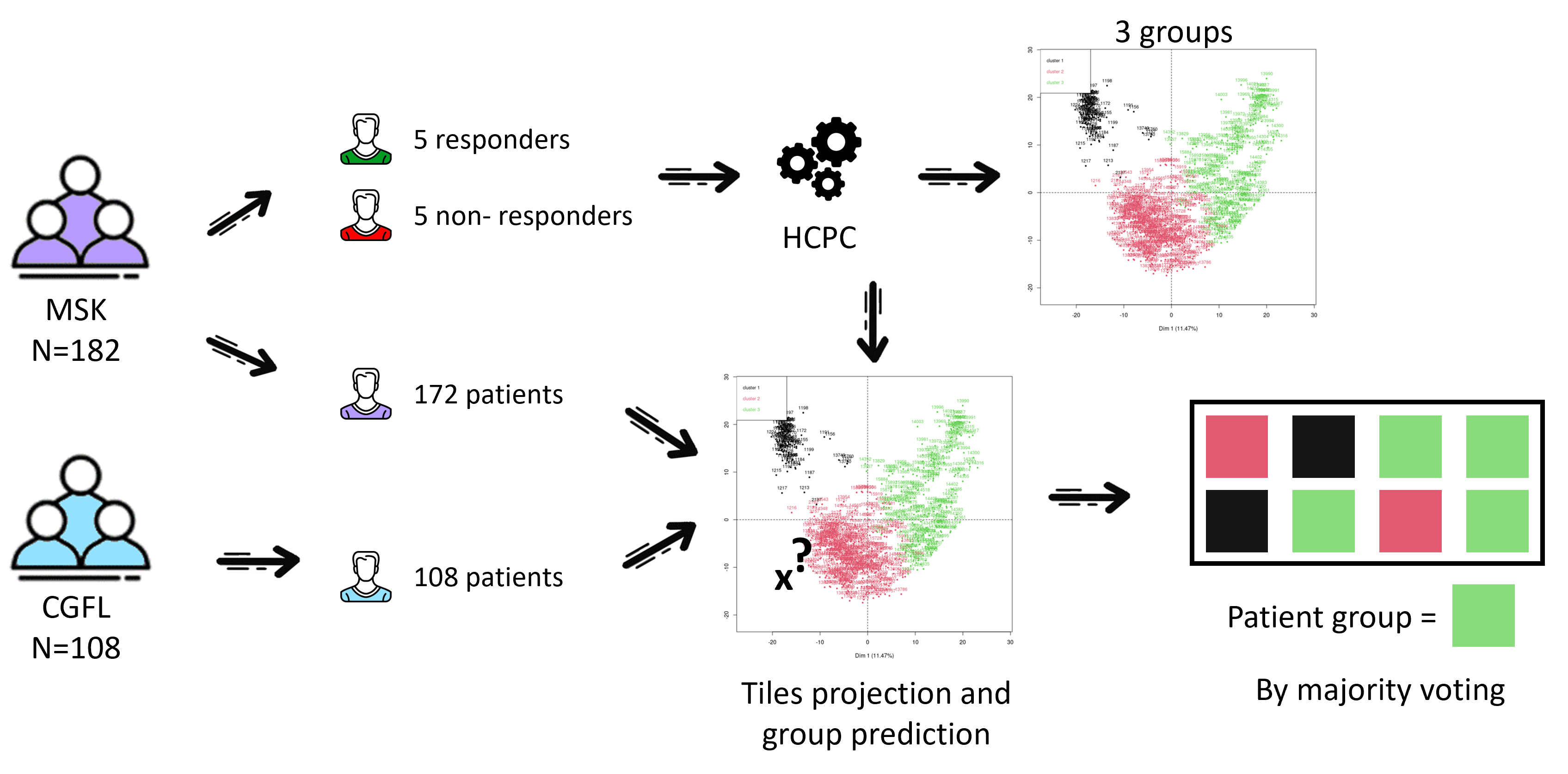

Supplement: Supplementary Figure 1 — Workflow of the tile-based clustering and classification. From the MSK cohort (n=182), 10 patients (five responders and five non-responders) were used to perform Hierarchical Clustering on Principal Components (HCPC), identifying two IHC-based clusters. Tiles from the remaining MSK patients (n=172) and the independent CGFL cohort (n=108) were then projected onto this reference space and assigned to the closest cluster, allowing patient-level group prediction. [file Image1.tif]
